# Acupuncture for the Treatment of Tension-Type Headache: An Overview of Systematic Reviews

**DOI:** 10.1155/2020/4262910

**Published:** 2020-03-19

**Authors:** Jinke Huang, Min Shen, Xiaohui Qin, Weichi Guo, Hui Li

**Affiliations:** ^1^The Second Clinical Medical College of Guangzhou University of Chinese Medicine, Guangzhou, China; ^2^Department of Standardization of Chinese Medicine, The Second Affiliated Hospital of Guangzhou University of Chinese Medicine (Guangdong Provincial Hospital of Chinese Medicine, Guangdong Provincial Academy of Chinese Medical Sciences), Guangzhou, China; ^3^Department of Neurology, The Second Affiliated Hospital of Guangzhou University of Chinese Medicine (Guangdong Provincial Hospital of Chinese Medicine, Guangdong Provincial Academy of Chinese Medical Sciences), Guangzhou, China; ^4^Engineering and Technology Research Center of Standardization of Traditional Chinese Medicine, Guangzhou, China

## Abstract

**Objectives:**

Because current evidence regarding the effectiveness of acupuncture for a tension-type headache (TTH) is controversial, we evaluated the reliability of the methodological quality and outcome measures of systematic reviews/meta-analyses (SRs/MAs) on acupuncture for TTH.

**Methods:**

We conducted a comprehensive literature search for SRs/MAs in major databases from the database's inception to September 2019. The Methodological Quality of Systematic Reviews 2 (AMSTAR-2) and the Grading of Recommendations, Assessment, Development, and Evaluation (GRADE) assessments were used to assess the methodological quality of the included reviews and the quality of evidence, respectively.

**Results:**

Eight reviews were included in the analysis. The AMSTAR-2 assessment results showed that the methodological quality of all included reviews was critically low. Thirty-six outcome measures were included in these reviews. The GRADE results showed that 25 (25/36, 69.4%) outcomes provided low- or very low-quality evidence, four (4/36, 11.1%) provided moderate-quality evidence, and seven (7/36, 19.4%) provided high-quality evidence. Descriptive analysis results showed that acupuncture treatment for TTH reduced headache frequency and severity.

**Conclusions:**

Acupuncture appears to be an effective treatment modality for TTH, but the credibility of the results is limited owing to the generally low methodological quality and evidence quality in the included SRs/MAs.

## 1. Introduction

A tension-type headache (TTH) is the most common primary headache in adults [1]. According to the 2013 Global Burden of Disease study, TTH has become the second most common chronic disease worldwide [2] and greatly affects patients' moods, daily work, and general life activities [3]. The TTH pathogenesis remains unclear but may be related to peripheral myofascial mechanisms (myofascial nociception) and central mechanisms (sensitization and inadequate endogenous pain control) [4]. The unclear TTH pathogenesis leads to headaches that are difficult to treat; the most common treatments include medication, physical therapy, and relaxation/cognitive therapy management [4]. Acupuncture is often used to treat headaches [5] and has been recommended by the European Federation of Neurological Societies as a complementary treatment option for TTH [6].

As sources of the highest level of evidence for evidence-based medicine, systematic reviews (SRs)/meta-analyses (MAs) have been widely used in recent years. A literature search yielded several published SRs/MAs on acupuncture, but their quality varied, and their results were highly controversial. To further synthesize the evidence, we composed an overview of the SRs [7].

## 2. Materials and Methods

The methodology for this overview was based on the Cochrane Handbook [8] and some high-quality methodological articles [9, 10].

### 2.1. Criteria for considering Reviews

#### 2.1.1. Types of Studies

SRs/MAs are based on randomized controlled trials (RCTs) of acupuncture for TTH. The language placed in any of the studies is limited to Chinese and English.

#### 2.1.2. Types of Participants

Participants in eligible studies were diagnosed with TTH, not limited by gender, age, ethnicity, time of onset, and source of the case.

#### 2.1.3. Types of Intervention

Treatment measures in the intervention group included various acupuncture therapies (e.g., acupuncture, electroacupuncture, auricular acupuncture, and body acupuncture) or acupuncture combined with other therapies. Treatments in the control group included comfort therapy (e.g., a placebo, sham acupuncture, or a blank control) or other therapies (e.g., medication therapy, Chinese medicine, or other nondrug therapies).

#### 2.1.4. Types of Outcomes

The included studies needed to report changes in at least one headache parameter include response rate, headache days, headache intensity, or headache duration.

### 2.2. Exclusion Criteria

Repeated publications, conference abstracts, comments, narrative reviews, and other overviews were excluded.

### 2.3. Search Strategy

PubMed, Embase, the Cochrane Library, the Web of Science, China National Knowledge Infrastructure (CNKI), Wanfang Database, Chongqing VIP, and Sino-Med (the Chinese database) were searched from their inceptions to September 2019. We searched the above databases using the following terminology: tension-type headache, acupuncture, systematic review, and meta-analysis.  Table 1 provides a search strategy for the PubMed database, and it is modified for different databases.

### 2.4. Data Collection and Extraction

The titles and abstracts of all searches were independently read by two reviewers (JK-H and M-S), and then the full texts of all potentially eligible articles were obtained. Two review authors independently examined these full-text articles in accordance with the inclusion criteria and selected eligible studies for inclusion in the review. Disagreements were resolved through consensus or by consulting an experienced and authoritative third reviewer (H-L).

Two reviewers (JK-H and M-S) extracted data independently using a standardized data extraction form. The following specific characteristics were extracted from each study: the first author, year of publication, country, number of included studies, sample size, treatment interventions, control interventions, quality assessment methods, outcomes, and main conclusions. The corresponding authors were contacted by email for missing information.

### 2.5. Quality Assessment

Two reviewers (JK-H and M-S) independently evaluated the quality of included reviews by using the Assessing the Methodological Quality of Systematic Reviews 2 (AMSTAR-2) [11], which contains 16 items, and seven of them are critical domains. Moreover, AMSTAR-2 proposes a four-level scheme (high, moderate, low, and critically low) for appraisers to rate the overall confidence in the results of a systematic review, and each item was evaluated using three evaluation options, “yes,” “partial yes,” and “no.”

The Grading of Recommendation, Assessment, Development, and Evaluation (GRADE) [12] was used to assess the quality of evidence by two reviewers (JK-H and M-S) independently. The following criteria were taken into account: risk of bias (that is study limitations), inconsistencies, indirectness, inaccuracy, and publication bias [13]. Any disagreements were resolved by consensus or discussion with the third author (H-L). Descriptive analysis was used for efficacy evaluation.

## 3. Results

### 3.1. Results on Literature Search and Selection

**Figure 1:**
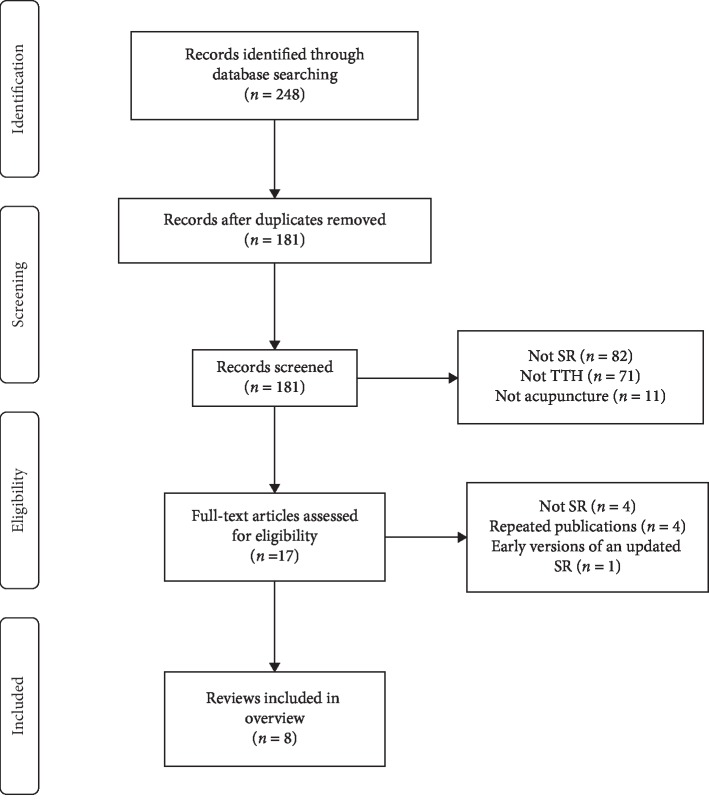
Flow diagram of the literature selection process.

A total of 248 citations were acquired from the electronic search; 67 duplicated articles were identified and excluded. 164 citations were excluded after screening the titles and abstracts for a variety of reasons, such as types of studies, interventions, and patients. Therefore, the full texts of the remaining 17 citations were retrieved for further evaluation. Nine publications were excluded for the following reasons: four were not SRs, four were repeated publications, and one was an early version of an updated SR. Thus, a total of 8 systematic reviews [14–21] were finally included in this overview. The selection process is recorded with a flow chart in Figure 1.

### 3.2. Characteristics of Included Reviews

The included reviews were published in the period from 2005 to 2018. Four SRs/MAs (50%) were published in Chinese and the remaining four (50%) were published in English. The number of RCTs included in the SRs/MAs varied widely, ranging from 5 to 25 studies, and the total participants ranged from 571 to 3916. The quality assessment scales of the original studies varied across the included SRs/MAs, five [15, 17, 19–21] used Jadad scale, and the remaining three [14, 16, 18] used Cochrane risk of bias criteria. The characteristics of the included reviews are summarized in Table 2.

### 3.3. Methodological Appraisal

**Figure 2:**
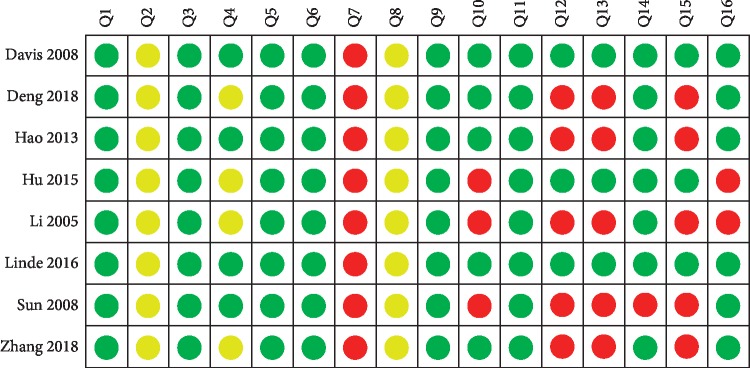
Summary of the AMSTAR-2 assessments.

**Figure 3:**
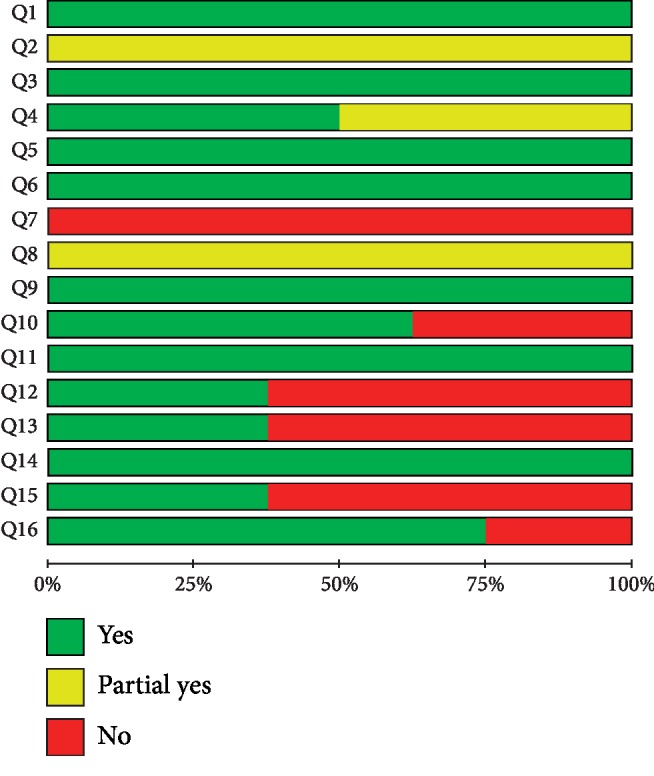
Graphical representation of the AMSTAR-2 assessments.

The quality of the included reviews was rated by AMSTAR-2. None of the included studies registered a protocol, which would affect the rigor of developing SRs/MAs. None of the included studies provided the list of excluded research literature, making it difficult to evaluate their practicality, and reduced their use value. Six reviews [14–17, 20, 21] had incomplete elements of the literature search strategy and did not provide the use of a specific search strategy; thus, we could not repeat the data or verify whether the search was complete. One review [16] searched only a Chinese database, thus easily missing studies in other databases. Five reviews [14–17, 19] did not search the gray literature, which may cause publication bias. Four reviews [14–17] omitted a conflict of interest statement; thus, there may have been a potential conflict of interest affecting the reported results. Overall, all included reviews had multiple critical domains that were unmet; these reviews were judged to be of very low quality. The results of the AMSTAR-2 assessment are given in Figures 2 and 3.

Green, yellow, and red in Figures 2 and 3 represent yes, partial yes, and no, respectively. Q1: Did the research questions and inclusion criteria for the review include the components of PICO? Q2: Did the report of the review contain an explicit statement that the review methods were established prior to the conduct of the review and did the report justify any significant deviations from the protocol? Q3: Did the review authors explain their selection of the study designs for inclusion in the review? Q4: Did the review authors use a comprehensive literature search strategy? Q5: Did the review authors perform study selection in duplicate? Q6: Did the review authors perform data extraction in duplicate? Q7: Did the review authors provide a list of excluded studies and justify the exclusions? Q8: Did the review authors describe the included studies in adequate detail? Q9: Did the review authors use a satisfactory technique for assessing the risk of bias (RoB) in individual studies that were included in the review? Q10: Did the review authors report on the sources of funding for the studies included in the review? Q11: If meta-analysis was performed did the review authors use appropriate methods for statistical combination of results? Q12: If meta-analysis was performed, did the review authors assess the potential impact of RoB in individual studies on the results of the meta-analysis or other evidence synthesis? Q13: Did the review authors account for RoB in individual studies when interpreting/discussing the results of the review? Q14: Did the review authors provide a satisfactory explanation for, and discussion of, any heterogeneity observed in the results of the review? Q15: If they performed quantitative synthesis, did the review authors carry out an adequate investigation of publication bias (small study bias) and discuss its likely impact on the results of the review? Q16: Did the review authors report any potential sources of conflict of interest, including any funding they received for conducting the review.

### 3.4. GRADE Evidence Quality Classification

Eight SRs/MAs included 36 outcomes related to the effectiveness of acupuncture for TTH. The results of GRADE evaluation showed that 25 (25/36, 69.4%) outcomes provided low- or very low-quality evidence, 4 (4/36, 11.1%) provided moderate-quality evidence, and 7 (7/36, 19.4%) provided high-quality evidence. Risk of bias (24/36, 66.7%) was the most common of the downgrading factors in the included SRs/MAs, followed by publication bias (20/36, 55.6%), imprecision (16/36, 44.4%), inconsistency (6/36, 16.7%), and indirectness (0/36, 0%). The results of the GRADE assessment are given in Table 3.

### 3.5. Outcomes and Efficacy Evaluation

#### 3.5.1. Response

Five reviews [14–18] analyzed the overall response rate to acupuncture for TTH. The pooled results indicated that the overall effectiveness of acupuncture for TTH differed among the systematic reviews.

Three reviews [15, 17, 18] reported the total effective rate of acupuncture versus sham acupuncture for TTH. The comprehensive results of one review [14] showed that the acupuncture group had a better overall effective rate than did the sham acupuncture control group at the end of treatment (RR = 1.25, 95% CI (1.08, 1.44), *P*=0.003 < 0.05), while another review [17] reported no difference (RR = 1.55, 95% CI (0.97, 2.47), *P*=0.07). Linde et al. [18] divided the follow-up time into four periods: 2 months, 3-4 months, 5-6 months, and 6 months after starting treatment; the second time window was the fourth week after the treatment ended. Their results showed that the acupuncture group had a better overall effective rate than did the sham acupuncture group for the 2-month (RR = 1.27, 95% CI (1.09, 1.48), *P*=0.0008) and 3-4-month (RR = 1.26, 95% CI (1.10, 1.45), *P*=0.003) periods; however, the two groups did not differ at 5-6 months (RR = 1.26, 95% CI (1.10, 1.45), *P*=0.02) or 6 months (RR = 1.17, 95% CI (1.02, 1.35), *P*=0.45).

Two reviews [14, 16] reported the effectiveness of acupuncture and nonacupuncture for treating TTH. Overall, acupuncture treatment was superior to nonacupuncture therapy such as those therapies used in western medicine (RR = 3.94, 95% CI (2.37, 6. 56), *P* < 0.00001).

#### 3.5.2. Headache Intensity

Seven reviews [14–18, 20, 21] analyzed the headache intensity of acupuncture for TTH. The pooled results indicated that headache intensity changes following acupuncture for TTH differed among the systematic reviews.

Five reviews [15, 17, 18, 20, 21] reported intensity changes in TTH after acupuncture versus sham acupuncture. One review [15] found that acupuncture significantly reduced VAS scores and improved headache symptoms in patients with TTH at the end of treatment (MD = 0.30, 95% CI (0.07, 0.52), *P*=0.009 < 0.05). However, another review [17] reported no significant difference in VAS scores between the acupuncture and sham acupuncture groups in patients' improvement at the end of treatment, within 2 months after treatment or 2 months after treatment (WMD = 0.55, 95% CI (−1.20, 0.09), *P*=0.44; WMD = 0.22, 95% (−0.87, 0.42), *P*=0.58; WMD = 0.65, 95% CI (−1.41, 0.11), *P*=0.63). Linde et al. [18] found a small statistical difference at 5-6 months after initiating treatment (SMD = 0.12, 95% CI (−0.35, −0.04), *P*=0.013). Similarly, Davis et al. [20] found no significant advantage in the acupuncture group compared with the sham acupuncture group in reducing headache intensity among patients during treatment (WMD = −7.24, 95% CI (−18.46, 3.99), *P*=0.21) but found a significant advantage in the acupuncture group at long-term follow-up (WMD = −3.64, 95% CI (−6.55, −0.73), *P*=0.01). Sun and Gan [21] followed TTH patients for 1 and 2 months after treatment, and the acupuncture group had a more statistically significant reduction in headache intensity than did the sham acupuncture group (WMD = −3.77, 95% CI (−7.00, −0.55), *P*=0.02; WMD = −3.66, 95% CI (−6.54, −0.79), *P*=0.01).

Three reviews [14–16] reported VAS score changes after acupuncture versus nonacupuncture treatment for TTH. VAS scores were more statistically significantly improved in patients with TTH after acupuncture treatment than after medication therapy (MD = 0.86, 95% CI (0.38, 1.33),*P*=0.00004; MD = –0.58, 95% CI (−0.63, −0.54), *P* < 0.00001). Additionally, acupuncture treatment improved the VAS scores more significantly than did the treatment with Chinese patent medicine (MD = 1.97, 95% CI (1.21, 2.72), *P* < 0.00001). However, the groups treated with acupuncture combined with traditional Chinese medicine did not significantly differ from those treated with western medicine alone (MD = 0.32, 95% CI (−0.45, 1.09), *P*=0.41).

#### 3.5.3. Headache Days

Four reviews [15, 18–20] analyzed the changes in headache days after acupuncture versus sham acupuncture contrast treatment. Zhang et al. [15] showed that the number of headache days after treatment was statistically significantly reduced in the acupuncture group (MD = 1.62, 95% CI (0.41, 2.82), *P*=0.009). Linde et al. [18] found that the number of headache days per month was significantly reduced in the acupuncture treatment group at 2 months, 3-4 months, and 5-6 months after initiating treatment (MD = −1.49, 95% CI (−2.58, −0.39), *P*=0.008; MD = −1.62, 95% CI (−2.69, −0.54), *P*=0.003; MD = −1.51, 95% CI (−2.59, −0.43), *P*=0.006). However, Hao et al. [19] found no statistically significant difference between the acupuncture and sham acupuncture groups at any time point. Davis et al. [20] found that although the acupuncture group did not significantly differ from the sham acupuncture group during the treatment period, the two groups differed significantly at 20–25 weeks after the treatment ended (WMD = −1.83, 95% CI (−3.01, −0.64), *P*=0.008).

#### 3.5.4. Headache Index

Zhang et al. [15] showed that acupuncture was superior to NSAIDs for treating TTH (MD = 4.81, 95% CI (3.55, 4.01), *P* < 0.00001) when a headache index was used as an evaluation index.

#### 3.5.5. Adverse Events

A review [18] showed no significant difference in adverse events between the acupuncture and sham acupuncture groups (OR = 1.26, 95% CI (0.60, 2.65), *P*=0.55) or between the acupuncture and nonacupuncture treatment groups (OR = 1.72, 95% CI (0.07, 42.81), *P*=0.74).

## 4. Discussion

A SR/MA overview is a comprehensive research method for reevaluating a comprehensive collection of SRs/MAs related to the same disease or health problem. A SR/MA overview enables more comprehensively integrating evidence, thus providing higher quality evidence for clinicians. Publication of SRs/MAs on acupuncture for TTH is increasing annually. The present analysis evaluated the methodological quality and quality of evidence from published SRs/MAs to provide an evidence-based assessment and objective summary of the effectiveness and safety of acupuncture for TTH. A literature search revealed that no overview of acupuncture for TTH has been published to date.

### 4.1. Summary of Evidence

Assessment of various aspects of the methodological quality of the included SRs/MAs using the AMSTAR-2 identified areas for common improvement. The evaluation results of the seven key items showed that none of the studies included a preliminary design protocol, which may result in a larger adjustment of the study process than expected, increasing the risk of bias and affecting the rigor of the systematic review. None of the included reviews provided a list of excluded studies, which may affect the reliability of the results. High-quality systematic reviews should provide a list of potentially relevant studies that do not meet the inclusion criteria and account for exclusion to guarantee transparency [22]. Of the studies analyzed, 75% provided only search keywords but no specific search strategy, and 62.5% did not adequately report or conduct a gray literature search. These factors likely contributed to generating publication bias and undermined the conclusion's reliability [23]. Of the systematic reviews, 62.5% did not consider the risk of bias among the included RCTs when the authors interpreted or discussed the study results, which may affect the authenticity of the final results. Deficiencies in these key items were the main reasons for the extremely low methodological quality evaluation results of the included SRs/MAs in this study. In addition, evaluation of the remaining nine items showed that the descriptions of the included studies in all systematic reviews were incomplete, such as the descriptions of the study subjects, interventions, control measures, and follow-up times. These incomplete original data may lead to heterogeneity. Of the studies, 37.5% did not report the funding source, and 75% did not declare whether the authors had conflicts of interest. These findings may impact the results because the results of business-funded studies might be biased towards the funder [24]. Of the systematic reviews, 62.5% did not assess the potential impact of the risk of bias of each included study on the results of evidence synthesis, and 12.5% did not specify the source of heterogeneity, which affected the rigor of the systematic reviews as the highest source of evidence. Future researchers should carefully follow the rules of the relevant items of the AMSTAR-2 and strictly control the study's methodological quality.

The quality of evidence included in the SRs/MAs was graded using the GRADE; most of the evidence was of low quality, indicating that the conclusions of the systematic review may differ from the true results and thus cannot provide a scientific basis for clinicians. Assessing the methodological quality of the original RCTs included in the SRs/MAs revealed that 66.7% of the evidence quality was downgraded because of the high risk of bias in the original RCTs: most of these RCTs only mentioned randomization but did not describe the randomization method; most did not conceal the allocation; only a few mentioned blinding, and most of these used only single blinding. Therefore, we believe the main factor leading to the degradation in evidence quality was the irrationality of the included studies in terms of implementing randomization, blinding, and allocation concealment. Although the particularities of acupuncture therapy can make blinding difficult to implement [25], well-designed and implemented RCTs are considered gold standards for evaluating interventions to minimize or avoid bias [26]. Future studies should take a more scientific approach to address the above issues to avoid the risk of bias. In addition, owing to the differences in the inclusion and exclusion criteria and literature search methods, there is also a risk of heterogeneity, precision, and publication bias.

In this overview, two SRs/MAs [17, 20] did not conclude positive findings, and both of them were published in the period from 2005 to 2008, meaning the available evidence may be lacking during that period. Instead, later studies drew a positive result about the efficacy of acupuncture for TTH.

### 4.2. Limitations

This overview has certain limitations. Above all, valuation of methodological quality and quality of evidence was a subjective process; different researchers had their own independent judgment on each factor, so the results may vary. Though this study had been independently evaluated and checked by two researchers, it may still be different from other studies. Following this, though the selection of the AMSTAR-2 for quality assessment is a strength of this study, it may also bring some insufficiency, because the 75% included SRs/MAs were published before the release of AMSTAR-2, so some authors may fail to follow the rules, which could partially result in low quality for assessment.

## 5. Conclusion

Acupuncture appears to be an effective treatment modality for TTH, but the methodological quality and evidence quality of the included studies were generally low, thus limiting the credibility of the results. Future studies should follow the relevant norms of the AMSTAR-2 and GRADE assessments as much as possible to further improve the study quality.

## Figures and Tables

**Table 1:** Search strategy for the PubMed database.

Query	Search term
# 1	Tension-type headache [mesh]
# 2	Tension-type headache∗[title/abstract] OR tension type headache∗[title/abstract] OR idiopathic headache∗[title/abstract] OR stress headache∗[title/abstract] OR tension headache∗[title/abstract] OR psychogenic headache∗[title/abstract]
# 3	#1 OR #2
# 4	Acupuncture [mesh]
# 5	Acupuncture [title/abstract] OR pharmacoacupuncture [title/abstract] OR acupotomy[title/abstract] OR acupotomies [title/abstract] OR pharmacopuncture[title/abstract] OR needle [title/abstract] OR needling [title/abstract] OR dry-needling[title/abstract] OR body-acupuncture [title/abstract] OR electro-acupuncture [title/abstract] OR electro-acupuncture [title/abstract] OR auricular acupuncture [title/abstract]
# 6	#4 OR #5
# 7	Meta-analysis as topic [mesh]
# 8	Meta-analysis [publication type]
# 9	Systematic review [title/abstract] OR meta-analysis [title/abstract] OR meta-analysis [title/abstract] OR meta-analyses [title/abstract] OR meta-analysis [title/abstract]
# 10	#7 OR #8 OR #9
# 11	#3 AND #6 AND #10

**Table 2:** Characteristics of the included reviews.

First author; year	Country	Trials (sample size)	Treatment intervention	Control intervention	Quality assessment tool	Overall conclusion
Deng [14] 2018	China	12 (933)	AT; AT + CPM; AT + MT	CPM; MT	Cochrane criteria	Acupuncture treatment of TTH has certain advantages.
Zhang et al. [15] 2018	China	10 (1071)	AT	SAT; NSAIDs	Jadad score	The acupuncture treatment is effective for TTH, and the therapeutic effect is better than NSAIDs.
Lin and Xiao [16] 2015	China	9 (598)	AT	MT	Cochrane criteria	The therapeutic effect of acupuncture on TTH has certain advantages compared with MT.
Li and Luo [17] 2005	China	13 (571)	AT	SAT; other physical therapies; MT	Jadad score	Comparing acupuncture with sham acupuncture and other treatments, current evidence cannot evaluate whether acupuncture is significantly effective for TTH.
Linde et al. [18] 2016	Germany	11 (2349)	AT	SAT; other physical therapies	Cochrane criteria	AT is effective for treating frequent episodic or chronic TTH.
Hao et al. [19] 2013	Australian	5 (838)	AT	SAT	Jadad score	AT stimulation mode, needle retention, and treatment frequency could be important factors contributing to the outcome of acupuncture for TTH.
Davis et al. [20] 2008	American	8 (896)	AT	SAT	Jadad score	The results suggest that AT compared with sham for TTH has limited efficacy for the reduction of headache frequency.
Sun and Gan [21] 2008	American	25 (3916)	AT	SAT; other physical therapies	Jadad score	AT is superior to SAT and MT in improving headache intensity, frequency, and response rate.

AT: acupuncture therapy; SAT: sham acupuncture therapy; NAT: nonacupuncture therapy; CPM : Chinese patent medicine; NSAIDs: nonsteroidal anti-inflammatory drugs; MT: medication therapy.

**Table 3:** Quality of evidence included systematic reviews with GRADE.

Author; year	Outcomes	Studies (participants)	Limitations	Inconsistency	Indirectness	Imprecision	Publication bias	Quality
Deng et al. [14] 2018	Response							
	AT versus SAT	12 (933)	−1①	0	0	0	−1⑤	Low
	VAS							
	AT versus MT	3(162)	−1①	0	0	−1④	−1⑤	Very low
	AT versus CPM	2 (145)	−1①	0	0	−1④	−1⑤	Very low
	AT + herbal versus MT	2 (166)	−1①	0	0	−1④	−1⑤	Very low

Zhang et al. [15] 2018	Response							
	AT versus SAT	4 (694)	−1①	0	0	0	−1⑤	Low
	AT versus NSAIDs	3 (168)	−1①	0	0	−1④	−1⑤	Very low
	VAS							
	AT versus SAT	4 (693)	0	−1②	0	0	−1⑤	Low
	AT versus NSAIDs	2 (102)	−1①	−1②	0	0	−1⑤	Very low
	Headache days							
	AT versus SAT	3 (754)	−1①	0	0	0	−1⑤	Low
	Headache index							
	AT versus NSAIDs	3 (168)	−1①	−1②	0	−1④	−1⑤	Very low

Lin and Xiao [16] 2015	Response							
	AT versus MT	9 (598)	−1①	0	0	0	−1⑤	Low
	VAS							
	AT versus MT	4 (236)	−1①	0	0	−1④	−1⑤	Very low

Li and Luo [17] 2005	Response							
	AT versus SAT (after treatment)	2 (48)	−1①	0	0	−1④	−1⑤	Very low
	VAS							
	AT versus SAT (after treatment)	3 (154)	−1①	0	0	−1④	−1⑤	Very low
	AT versus SAT (within 2 months)	3 (152)	−1①	0	0	−1④	−1⑤	Very low
	AT versus SAT (more than 2 months)	2 (103)	−1①	0	0	−1④	−1⑤	Very low

Linde et al. [18] 2016	Response							
	AT versus SAT (within 2 months)	4 (1093)	0	0	0	0	0	High
	AT versus SAT (3-4 months)	4 (703)	0	0	0	0	0	High
	AT versus SAT (5-6 months)	4 (723)	0	0	0	0	0	High
	AT versus SAT (more than 6 months)	1 (30)	0	0	0	−1④	0	Moderate
	Headache days							
	AT versus SAT (within 2 months)	4 (682)	0	0	0	0	0	High
	AT versus SAT (3-4 months)	4 (653)	0	0	0	0	0	High
	AT versus SAT (5-6 months)	4 (670)	0	0	0	0	0	High
	Headache intensity							
	AT versus SAT (3-4 months)	4 (655)	0	0	0	−1④	0	Moderate
	AT versus SAT (5-6 months)	4 (670)	0	0	0	0	0	High
	Safety							
	AT versus SAT	3 (277)	0	−1②	0	−1④	0	Low
	AT versus NAT	1 (207)	0	−1②	0	−1④	0	Low

Hao et al. [19] 2013	Headache days							
	AT versus SAT (after treatment)	5 (729)	−①	−1②	0	0	0	Low
	AT versus SAT (within 3 months)	3 (510)	−①	0	0	−1④	0	Low
	AT versus SAT (more than 3 months)	4 (684)	−①	0	0	−1④	0	Low

Davis et al. [20] 2008	Headache days							
	AT versus SAT (during treatment)	5 (729)	−1①	0	0	0	−1⑤	Low
	AT versus SAT (20–25 weeks)	4 (723)	−1①	0	0	0	−1⑤	Low
	Headache intensity							
	AT versus SAT (during treatment)	3 (199)	−1①	0	0	0	−1⑤	Low
	AT versus SAT (20–25 weeks)	4 (713)	−1①	0	0	0	−1⑤	Low

Sun and Gan [21] 2008	Headache intensity							
	AT versus SAT (1 month later)	6 (762)	−1①	0	0	0	0	Moderate
	AT versus SAT (2 months later)	4 (681)	−1①	0	0	0	0	Moderate

AT: acupuncture therapy; SAT: sham acupuncture therapy; NAT: nonacupuncture therapy; CPM : Chinese patent medicine; NSAIDs: nonsteroidal anti-inflammatory drugs; MT: medication therapy; VAS: visual analog scale; ①: the design of the experiment with a large bias in random, distributive hiding or blind; ②: the confidence interval overlaps less, the heterogeneity test P is very small, and the I^2^ is larger; ③: confidence interval is not narrow enough; ④: funnel graph asymmetry; ⑤: fewer studies are included and there may be greater publication bias.

## Data Availability

The datasets used in the present review are available from the corresponding author on reasonable request.

## Abbreviations

TTH: Tension-type headache
SR: Systematic review
MA: Meta-analysis
AMSTAR-2: Assessing the Methodological Quality of Systematic Reviews 2
GRADE: Grading of Recommendations, Assessment, Development, and Evaluation
RCTs: Randomized clinical trials
VAS: Visual analog scale
CNKI: China National Knowledge Infrastructure
NSAIDs: Nonsteroidal anti-inflammatory drugs.
